# Long-Term Safety from the Raltegravir Clinical Development Program

**DOI:** 10.2174/157016211794582650

**Published:** 2011-01

**Authors:** Hedy Teppler, Deborah D Brown, Randi Y Leavitt, Peter Sklar, Hong Wan, Xia Xu, Fabio Lievano, Heidi P Lievano, T Christopher Mast, Bach-Yen T Nguyen

**Affiliations:** Merck Research Laboratories, North Wales, PA, USA

**Keywords:** Raltegravir, integrase inhibitor, long-term safety, BENCHMRK, STARTMRK.

## Abstract

**Background:**

Raltegravir has demonstrated potent and durable efficacy and a favorable safety profile in 3 phase III studies in treatment-naïve and treatment-experienced patients with HIV-1 infection. This manuscript provides a review of the raltegravir safety profile using data from these and other studies in the clinical development program.

**Methods:**

Comprehensive 96-week safety data from STARTMRK (raltegravir versus efavirenz, each with tenofovir/emtricitabine) and BENCHMRK (raltegravir versus placebo, each with optimized background therapy) are summarized. A cumulative meta-analysis of raltegravir 400 mg bid was conducted across the entire development program.

**Results:**

In STARTMRK, drug-related adverse events (AEs) occurred less frequently with raltegravir than efavirenz. In BENCHMRK, the most common drug-related AEs occurred at generally similar frequencies in both groups. Drug-related serious AEs were uncommon. Rash was observed in raltegravir-treated patients at a higher frequency than placebo but a lower frequency than efavirenz. Depression and immune reconstitution inflammatory syndrome occurred at similar rates for raltegravir and comparators. Isolated elevations of creatine kinase were more common with raltegravir than placebo but occurred without clinical manifestations. The frequency of aminotransferase elevations was greater in patients with viral hepatitis co-infection, but similar in the raltegravir and comparator groups. The relative risk (95% CI) of cancer was 0.75 (0.30, 1.91) indicating no difference between raltegravir and comparator. Overall trends in the cumulative meta-analysis were similar to those observed in the phase III studies.

**Conclusions:**

Long-term data from the phase III clinical trials demonstrate that raltegravir was generally well-tolerated in both treatment-naïve and treatment-experienced patients with HIV infection.

## INTRODUCTION

Treatment with a combination of highly active antiretroviral agents (HAART) is the standard of care for patients with human immunodeficiency virus (HIV) infection, yet is often complicated by the development of antiretroviral resistance, toxicity and adverse experiences (AEs) associated with one or more agents, underlying medical conditions, drug interactions, and personal preference of patients and care-providers. New therapies are needed.

Raltegravir is an HIV integrase inhibitor that blocks HIV replication by specific inhibition of proviral DNA-strand transfer, a mechanism of action distinct from that of other approved classes of antiretroviral drugs [1]. Raltegravir has potent *in vitro* activity against HIV-1 strains susceptible or resistant to other antiretroviral drug classes^1^. Initially approved in the United States and other countries in 2007 to be used in combination therapy for the treatment of HIV infection in patients failing previous HAART, raltegravir is now approved for use in both treatment-naïve and treatment-experienced patients, based on the demonstration of potent and durable efficacy, good tolerability and a favorable safety profile in 3 phase III studies: Protocol 021 (known as STARTMRK) in treatment-naïve patients [2,3], and Protocols 018 and 019 (known as BENCHMRK-1 and BENCHMRK-2, respectively) in treatment-experienced patients [4-6].

This manuscript provides a review of the safety profile of raltegravir, summarizing the comprehensive 96 week safety data from these phase III trials. A number of conditions of interest were identified for specific review either based on data from the raltegravir clinical trials, or because they have been reported as post-marketing AEs for raltegravir or were associated with other antiretroviral agents. These events include serious rash/Stevens Johnson syndrome (SJS), lipodystrophy, immune reconstitution inflammatory syndrome (IRIS), increase in liver enzymes and related clinical hepatic events, elevations of creatine phosphokinase (CK) with clinical manifestations including rhabdomyolysis and myopathy, and depression including suicidal ideation and behaviors. Malignancies are also specifically examined because the initial analysis of the BENCHMRK data suggested an imbalance in the occurrence of cancers; however, review of subsequent data does not suggest an increased risk of malignancy with raltegravir treatment. A cumulative meta-analysis across the entire development program was also conducted to examine the comprehensive safety of the 400 mg bid raltegravir dose. In aggregate, the long-term data from the phase III clinical trials demonstrates that raltegravir was generally well-tolerated in both treatment-naïve and treatment-experienced HIV-infected patients.

## MATERIAL AND METHODS

STARTMRK is a multicenter, double-blind (DB), randomized, active-controlled study to evaluate the safety and antiretroviral activity of raltegravir versus efavirenz, each in combination with tenofovir/emtricitabine, in treatment-naïve HIV-infected patients [2,3]. BENCHMRK-1 and -2 are identical multicenter, double-blind, randomized, placebo-controlled studies to evaluate the safety and antiretroviral activity of raltegravir in combination with an optimized background therapy (OBT), versus optimized background therapy alone, in HIV-infected patients with documented 3-class resistance; both studies included an open-label treatment option for patients with virologic failure [4-6]. BENCHMRK data presented here focus on the DB treatment phase and are shown for the 2 studies combined. An independent data and safety monitoring board periodically monitored the blinded safety and efficacy results of all 3 trials. Because of the difference in baseline characteristics, especially HIV disease stage, concurrent conditions and medications in the two populations studied, the safety data are presented separately for treatment-naïve and treatment-experienced populations except for the malignancy analysis.

AE terms were coded using the Medical Dictionary for Drug Regulatory Affairs (MedDRA, Version 12.0). Investigators were required to assess each AE for both intensity and causal relationship to study regimen. Intensity was assessed as mild, moderate, or severe based upon degree of interference with routine activities. Causality was assessed based on overall investigator judgment as definitely, probably, possibly, probably not, or definitely not related to raltegravir or comparator (efavirenz or placebo) either alone or in combination with other background therapy (tenofovir/emtricitabine or OBT). AEs with causality assessment of possible, probable, or definite were considered "drug-related." Serious AEs (SAEs) were those which met any of the following criteria: resulted in death, led to or prolonged hospitalization, was a persistent or significant disability, was considered life threatening, a congenital anomaly (in the offspring of patient following prenatal exposure to raltegravir), a cancer, an overdose, or an "other important medical event" in the judgment of the investigator.

The following safety issues were identified from clinical trials, post-licensure reports, or as general concerns for antiretroviral agents: rash, SJS, depression including suicidal ideation and behaviors, IRIS, lipodystrophy, elevations of CK with clinical manifestations including rhabdomyolysis and myopathy, and increase in liver enzymes and related clinical hepatic events. For the assessment of these selected safety issues, all related specific MedDRA terms present in the database were included; the list of specific terms is provided in the respective sections. Frequencies of AEs (both all causality and drug related) are provided for all populations. Exposure-adjusted rates are provided for the BENCHMRK studies only.

Laboratory abnormalities were graded using Division of AIDS (DAIDS) toxicity criteria for the laboratory test of interest, identifying changes that represented a worsened grade from baseline, as patients were permitted to enroll with substantial baseline laboratory abnormalities. Baseline values were those obtained at the baseline study visit before initiation of study therapy. In the BENCHMRK studies, all clinical AIDS defining conditions (ADC) were reported as submitted by investigators for safety analysis; these events were submitted for blinded adjudication for separate consideration of confirmed ADCs as an efficacy parameter [4-6].

Exposure-adjusted rates are provided for the BENCHMRK clinical AEs and laboratory data because of unbalanced attrition resulting from higher rates of virologic failure in the placebo group; exposure was 708 person-years (PYR) for raltegravir and 244 PYR for placebo at the 96 week analysis. Exposure-adjusted rates provide the number of events (clinical AEs or laboratory abnormalities) per 100 PYR of exposure, using a crude population-based exposure adjustment with fixed exposure time; STARTMRK data were not exposure-adjusted since the study periods were balanced between the 2 treatment groups.

Since patients with active hepatitis B or C co-infection were permitted to enroll, this review of safety also includes specific examination of hepatic safety in co-infected patients.

The analysis of malignancy combines data from all 3 studies and uses all available DB data, including data beyond week 96 for some patients. Time-at-risk for patients with cancer in the DB phase is from randomization to first cancer diagnosis; time at risk for patients without cancer in the DB phase is from randomization to data cut-off (if continuing) or to 14 days after study discontinuation. Exposure-adjusted rate of cancer was the number of patients with cancer per 100 PYR. Relative risk (RR) and associated exact 95% CI were calculated to compare raltegravir with comparator during the DB phase.

A cumulative meta-analysis was also conducted to examine the comprehensive safety of the 400 mg bid raltegravir dose across the entire development program. This analysis includes all available DB data from Merck studies in HIV-infected adults that have been unblinded and presented or published: Protocols 004, 005, 032 and 033, in addition to 96 week data from the phase III STARTMRK and BENCHMRK studies described above. Protocol 004 is a dose-ranging phase II study of raltegravir versus efavirenz (both with lamivudine and tenofovir) in treatment naïve individuals; data up to week 144 are included 2. Protocol 005 is a dose-ranging phase II study of raltegravir versus placebo (both with OBT) in highly treatment-experienced patients [7]; 24-48 weeks of DB data are included. Protocols 032 and 033 (SWITCHMRK studies) are identical studies of raltegravir 400mg bid versus continued lopinavir/ritonavir (both with stable background regimens) in patients stably suppressed on a lopinavir/ritonavir regimen; DB data through week 24 are included [8]. A similar analytical approach was used for the cumulative meta-analysis for both AEs and treatment-emergent laboratory abnormalities. The focus was combined data from all patients who received raltegravir 400mg versus comparator in DB phase. Crude exposure adjustment was used to address the different durations of therapy in the raltegravir versus comparator groups. Details can be found in Supportive/Supplementary Material.

## RESULTS

### Phase III Studies

#### Demographics

Table **1** presents the demographic distribution of the treatment groups. The STARTMRK population had a greater proportion of non-white race/ethnicity, was younger, and had a lower proportion of males than the BENCHMRK population. Hepatitis B or C co-infection was present in approximately 6% of patients per group in STARTMRK and in approximately 16% per group in BENCHMRK.

#### Clinical Adverse Experiences

Table **2** displays clinical AEs for the STARTMRK and BENCHMRK studies, including frequencies of various categories of clinical AEs: overall, drug-related, serious, serious drug-related, death, discontinuations due to an AE, discontinuations due to a drug-related AE, the most frequent clinical AEs (reported in at least 10% in any group) irrespective of causality, and the most frequent drug-related AEs (reported in at least 2% in any group).

In STARTMRK, drug-related AEs occurred less frequently in the raltegravir group compared to the efavirenz group (p-value <0.001). For the various other categories of AEs, frequencies were generally similar between the raltegravir and efavirenz groups. In BENCHMRK, AEs of all categories occurred at similar frequencies between the raltegravir and placebo groups; after adjustment for exposure, the rates of AEs for raltegravir were in general lower than for placebo.

In STARTMRK, the most notable difference was for the specific AE of dizziness: 8.2% in the raltegravir group compared to 36.9% in the efavirenz group; most of these reports were considered drug-related: 6.0% *vs* 34.0%, respectively. Otherwise, the most common AEs in both treatment groups, irrespective of causality, were diarrhea and headache. Diarrhea was reported at a higher frequency in the efavirenz group; frequencies of headaches were similar in both groups. The most common drug-related AEs of any intensity were diarrhea, nausea, fatigue, headache, somnolence, abnormal dreams, insomnia, and rash, all of which were reported at higher frequencies in the efavirenz group than in the raltegravir group.

In the BENCHMRK studies, the most common AEs irrespective of causality were diarrhea, nausea, upper respiratory tract infection, and headache, which occurred at similar frequencies in the raltegravir and placebo groups. The most common drug-related AEs of any intensity were diarrhea, nausea, fatigue, and headache and these occurred at generally similar frequencies in both groups. Exposure-adjusted rates were generally lower for these AEs in the raltegravir group compared with the placebo group.

In all phase III trials, SAEs considered drug-related (possibly, probably or definitely) were uncommon, even in treatment-experienced patients with advanced disease. Drug-related SAEs (number of patients, study day of onset) in the raltegravir group in STARTMRK were immune reconstitution syndrome (2, d. 7 and d. 29), mental disorder (1, d. 76), suicide attempt (1, d. 510), and anemia (1, d. 512). Only the SAE of mental disorder led to discontinuation of therapy. In the BENCHMRK studies, drug-related SAEs in the raltegravir group were genital herpes (1, d. 134), hepatitis (1 pt, 2 occurrences, d. 30 and d. 102), gastritis (1 pt, 2 occurrences, d. 23 and d. 211), renal failure (1, d. 25), and accidental overdose (1, d. 233). The case with hepatitis was a flare of pre-existing hepatitis B, and the second occurrence resulted in discontinuation from study. The case with renal failure was considered most likely related to tenofovir in OBT (and possibly related to raltegravir) and this patient also discontinued from the study. The accidental overdose was a dosing error (patient consumed twice the recommended dose) and was not associated with any clinical AEs.

#### Treatment Emergent Laboratory Abnormalities

As displayed in Table **3**, treatment-emergent Grade 3 or 4 abnormalities were uncommon in the STARTMRK treatment-naïve population, and were generally seen in the clinically more advanced BENCHMRK study population. The most common Grade 3 and 4 lab abnormalities (occurring in at least 4%) were absolute neutrophil count, fasting total and LDL-cholesterol, fasting triglycerides, alanine aminotransferase, aspartate aminotransferase, and CK, which were reported at similar frequencies between the groups within each study and are examined further in the section Detailed Review of Specific Safety Issues.

In the BENCHMRK studies, the frequency of patients with Grade 3 or 4 lipid values was slightly higher in the raltegravir group compared to the placebo group. However, the differences are small and are difficult to interpret, as many patients had pre-existing lipid disorders (44.8% in the raltegravir group and 43.9% in the placebo group), and lipid-lowering agents were frequently used as prior and concomitant medications (data not shown). In order to address this confounding factor, a pre-specified analysis was conducted in STARTMRK and demonstrated minimal changes in lipid profile with raltegravir [2,3].

#### Detailed Review of Specific Safety Issues

Selected safety issues identified from clinical trials, post-licensure reports, or as general concerns for antiretroviral agents were examined and are summarized below.

##### Rash and Stevens-Johnson Syndrome (SJS)

In STARTMRK, rash occurred more frequently in the efavirenz group compared to the raltegravir group. In the BENCHMRK studies, the frequency of rash overall was higher in the raltegravir group compared to the placebo group, but drug-related rash occurred at comparable frequencies. Most cases of rash were mild to moderate. There were no serious AEs of rash and no cases of SJS in either population, and no patients discontinued raltegravir due to rash (Table **4**).

In addition to the general review of rash, rash in the presence of darunavir was of interest because its common occurrence led to the premature termination of a phase I study in uninfected subjects in which raltegravir and darunavir were co-administered^3^, and due to the frequent use of darunavir in OBT in the phase III studies [5,6]. In the BENCHMRK studies, the frequency of rash in patients taking darunavir was slightly higher in the raltegravir group compared to the placebo group, while drug-related rash occurred at comparable frequencies. Exposure-adjusted rates of rash showed similar trends (Table **4**).

##### Depression and Suicidality

In STARTMRK, depression/suicidality occurred at similar frequencies for raltegravir and efavirenz. In the BENCHMRK studies, the frequency of depression/suicidality was lower than observed in the STARTMRK study and was similar in the raltegravir and placebo groups. SAEs of depression/suicidality were uncommon in both populations, and there were no discontinuations from the raltegravir group due to these events. Exposure-adjusted rates of depression/suicidality were similar overall and low for drug-related events (Table **4**).

##### Immune Reconstitution Inflammatory Syndrome (IRIS)

In STARTMRK, IRIS occurred at similar frequencies in the raltegravir and efavirenz groups, and infrequently as an SAE; one raltegravir-treated patient discontinued due to IRIS. In the BENCHMRK studies, IRIS was reported at low and similar frequencies in the raltegravir and placebo groups; exposure-adjusted rates were similarly low (Table **4**).

##### Lipodystrophy

In STARTMRK, clinical AEs of lipodystrophy were uncommon. DEXA measurement showed minimal gains in body fat (Table **5**), with no patterns of fat loss over 96 weeks, suggesting that raltegravir is not associated with abnormal patterns of fat redistribution (lipodystrophy or lipoatrophy) over this period of observation. In the BENCHMRK studies, lipodystrophy occurred somewhat more often in the raltegravir group than the placebo group (Table **4**), but this observation is confounded by high proportions of lipodystrophy-related diagnoses at baseline; 44.8% in the raltegravir group and 43.9% in the placebo group, likely reflecting advanced HIV disease and substantial prior exposure to protease inhibitors.

##### Increase in Creatine Phosphokinase (CK) with Clinical Manifestations, Myopathy, and Rhabdomyolysis

In the STARTMRK study, there was one (0.4%) serious clinical AEs of myopathy in the raltegravir group, which the investigator considered not drug-related. There were no reports of myositis or rhabdomyolysis; CK was not routinely monitored. In the BENCHMRK studies, CK was routinely monitored and treatment-emergent Grade 3 or 4 CK values occurred more frequently for raltegravir than placebo. Exposure-adjusted rates were also somewhat higher for raltegravir. There was a single (0.2%) report of myositis with an associated Grade 2 CK value in the raltegravir group, which the investigator judged as related to ritonavir (in OBT), and one (0.1%) report of myopathy in the placebo group which was considered not drug-related by the investigator. There were no reports of rhabdomyolysis in either group. There were no raltegravir discontinuations related either to the CK elevations or the clinical AEs (Table **4**).

##### Hepatic Safety: Increase in Liver Enzymes (and Related Clinical Hepatic Events)

In the STARTMRK study, treatment-emergent Grade 3 or 4 elevations of alanine aminotransferase (ALT) and aspartate aminotransferase (AST) respectively, occurred in 1.8% and 2.8% of patients in the raltegravir group compared to 2.5% for both parameters in the efavirenz group. ALT/AST elevations did not limit raltegravir therapy; there were 3 discontinuations in the efavirenz group. Grade 3 and 4 bilirubin elevations were uncommon (Table **6**).

In the BENCHMRK studies, treatment emergent Grade 3 or 4 elevations of ALT and AST respectively, occurred in 5.2% and 4.8% of patients in the raltegravir group compared to 3.8% and 4.2% in the placebo group. One patient in the raltegravir group discontinued therapy due to ALT/AST elevation (considered not drug-related). The exposure-adjusted rates per 100 PYR for Grade 3 or 4 ALT and AST were, respectively, 3.4 and 3.1 in the raltegravir group compared to 3.7 and 4.1 in the placebo group. Grade 3 or 4 bilirubin elevations were uncommon (Table **6**) and mostly associated with atazanavir use.

In all 3 studies, clinical hepatic AEs were uncommon and occurred at similar frequencies in the raltegravir and comparator groups. In the BENCHMRK studies, serious hepatic events were infrequent and none resulted in death; one patient in the raltegravir group discontinued due to an AE of hepatitis (Table **6**).

Review of the safety profile of raltegravir was also performed in the subgroup of patients co-infected with hepatitis B and/or C, which was present in approximately 6% of STARTMRK and 16% of BENCHMRK-1 patients.

In general, Grade 3 or 4 aminotransferase elevations were more common in co-infected patients in all treatment groups, compared to patients without co-infection, but were not associated with Grade 3 or 4 bilirubin elevations. Clinical hepatic events were very uncommon, were not serious and did not limit therapy.

##### Malignancies

**Fig. (1):**
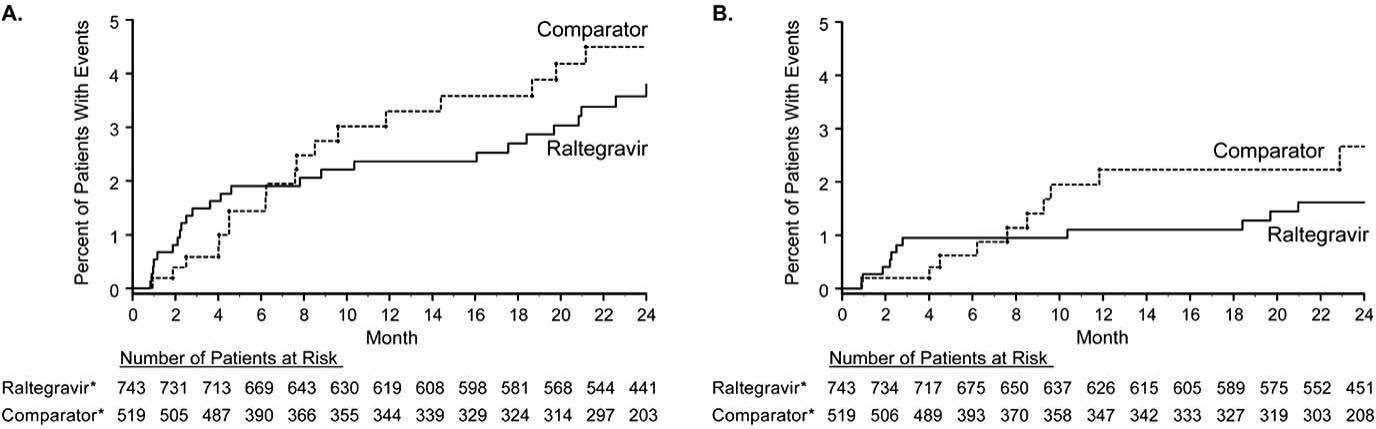
Kaplan-Meier estimates of time to malignancy event in the Phase III studies (Protocols 018, 019, and 021) using a broad cancer definition (Panel **A**) and the NCI/ACS definition (Panel **B**). The NCI/ACS definition does not include recurrence, carcinoma in situ, or non-melanoma skin events. *All patients also received OBT (in the BENCHMRK studies) or tenofovir with emtricitabine (in the STARTMRK study).

The present analysis of malignancies was performed consistently with previous analyses [4] and employed two case definitions. For the double-blind comparison, exposure-adjusted rates per 100 PYR of cancer for the 3 studies were 0.96 for raltegravir and 1.28 for comparator (efavirenz or placebo), resulting in a RR (95% CI) of 0.75 (0.30, 1.91) using the National Cancer Institute/American Cancer Society (NCI/ACS) definition. Using a broader definition that includes recurrences, carcinomas in situ, and non-melanoma skin cancers (such as basal cell and squamous cell carcinomas), the rates per 100 PYR were 2.02 and 2.33, respectively, resulting in a RR of 0.87 (0.46, 1.67). Similar results were obtained when the open-label phases of the studies were included (data not shown). Using time-to-event analysis, the occurrence of cancer events for raltegravir using either case definition diminished after the first 3 months of study and remained relatively flat compared to the comparator group (Fig. **1**). The 96-week data indicate the risk of cancer is similar for raltegravir and comparator groups whether all cancers or only NCI/ACS events are included, and there is no specific cancer risk associated with raltegravir treatment.

### Meta-Analysis

The cumulative meta-analysis included 1771 PYR of raltegravir 400mg BID exposure (over 2.5 fold greater compared with the STARTMRK and BENCHMRK studies alone). Overall trends for both number (%) and exposure-adjusted rates (1771 PYR for raltegravir *vs* 981 PYR for comparator) for all-causality, drug-related, serious, and serious drug-related adverse experiences (Table **7**), as well as treatment-emergent laboratory abnormalities (Table **8**), were broadly similar to those observed in the phase III studies. No new safety concerns for raltegravir were identified. Further details of the meta-analysis can be found in Supportive/Supplementary Material.

## DISCUSSION

Raltegravir has demonstrated a favorable safety profile in addition to its potent efficacy based on data from comparative phase III trials against efavirenz (with background of tenofovir and emtricitabine) in treatment-naïve patients [2,3] and against placebo (with OBT) in treatment-experienced patients [4-6]. Long-term data from the phase II studies in treatment-naïve and treatment-experienced populations, which include data to week 144, show a similar safety profile [9,10]^2, 4^.

Rash is frequently observed with several antiretroviral and other agents commonly used in the treatment of HIV infection [11-14]. In clinical trials, rash was observed in raltegravir-treated patients at a higher frequency than placebo but at a lower frequency than efavirenz. The incidence of rash was also higher among patients taking raltegravir and darunavir than in patients taking raltegravir without darunavir or darunavir without raltegravir. In general, rash was reported as mild to moderate and did not lead to discontinuation of raltegravir. It is important to note that raltegravir is only administered in combination with other antiretroviral agents, thus the reports of rash are confounded by concomitant antiretroviral and often other therapies, many known to be associated with rash. A number of antiretroviral agents have been associated with SJS [15]. This event was added to the raltegravir labeling after several post-marketing reports of SJS were received, even though the reports were confounded by the use of concomitant medications. There were no cases of SJS in the raltegravir phase III studies.

Depressive disorders appear to be more common in persons with HIV infection than in the general population [16]. Neuropsychiatric symptoms have been associated with efavirenz, particularly in the short term after initiation of therapy [17-22]. Depression, including suicidal thoughts and behaviors, was added to the raltegravir product labeling after identification of several post-marketing cases, including those reported in the literature [23]. In the phase III studies of raltegravir, depression occurred at generally similar rates between raltegravir and comparator, and one suicide attempt was reported.

IRIS has been reported with multiple HAART regimens [24-30] and is often associated with a specific diagnosis of an opportunistic infection or malignancy. Reports of IRIS in patients receiving raltegravir are consistent with what has been reported with other classes of antiretroviral agents.

Lipodystrophy is a common metabolic complication of antiretroviral therapy [31-38]. The incidence of lipodystrophy in the raltegravir phase III trials was low in treatment-naïve patients overall. Analysis of body fat content by dual energy x-ray absorptiometry (DEXA) scanning in a subset of patients showed similar degrees of fat gain through 96 weeks for each treatment group, with a greater increase in trunk than limb adiposity [3]. Also, raltegravir had minimal effect on serum lipid levels in treatment-naïve patients [2,3]. In treatment-experienced patients, lipodystrophy occurred at generally similar rates in the raltegravir and placebo groups, but these rates are confounded by the background prevalence of lipodystrophy (approximately 44-45% in both groups) and frequent concomitant use of other classes of antiretroviral therapies for which lipodystrophy/fat maldistribution have been reported.

Elevations of CK have been seen in clinical trials at a higher incidence for raltegravir than placebo; however, these were generally isolated laboratory findings, without clinical manifestations and did not lead to discontinuation. Although not seen in the comparative phase III studies, there have been several reports of rhabdomyolysis in expanded access programs for raltegravir, as well as in the post-marketing environment, including some reports in patients with confounding factors, such as concomitant use of medications known to be associated with this condition, including HMG-CoA reductase inhibitors and fibric acid derivatives. Rhabdomyolysis is included in product labeling for raltegravir.

Increased liver enzymes and, to a lesser degree, related clinical hepatic events, are common complications of antiretroviral and other therapies in HIV infected individuals; furthermore, interpretation may be confounded by the common occurrence of dual infection with HIV and hepatitis B or C virus. In clinical trials, aminotransferase elevations occurred at modest and similar rates in raltegravir and comparator groups. While these abnormalities were more frequent in patients with (than without) viral hepatitis co-infection, they occurred similarly in the raltegravir and comparator groups. Aminotransferase abnormalities were not associated with unexplained hyperbilirubinemia or clinical hepatic toxicity, and rarely limited therapy. Thus, raltegravir does not appear to be associated with important hepatotoxic effects, including in patients with hepatitis B or C co-infection.

Malignancies have been closely monitored since early in the development program after a possible signal was identified with slight excess in cancers observed in the BENCHMRK studies at the first interim analysis of partial data. With additional follow-up in subsequent analyses, the data demonstrate a RR (95% CI) of 0.75 (0.30, 1.91) indicating no difference in cancer risk between raltegravir and comparator. Possible explanations for the early, transient signal include a premature assessment of limited data, and the occurrence of IRIS acting to bring subclinical malignancies to early clinical detection in the setting of prompt virologic suppression and CD4 cell recovery. Two observational cohort studies have begun which will further assess this issue in the post-licensure environment.

This paper has presented all available comparative phase III data for raltegravir. The data have some limitations: in the BENCHMRK studies in highly treatment-experienced patients, the patient year exposure was 2-3 fold higher for raltegravir than comparator due to frequent virologic failure in the placebo group, resulting in retention of patients with the most advanced disease in the raltegravir group. Provision of both unadjusted frequencies and exposure adjusted rates for BENCHMRK data were an effort to offer an unbiased perspective on the occurrence of early versus late adverse experiences. Women represented <20% of patients enrolled in these phase III studies. Other limitations include the defined inclusion/exclusion criteria used in the context of clinical trials. However, these studies allowed enrollment of patients with advanced HIV disease, substantial laboratory abnormalities, active hepatitis B or C co-infection, and stable prior cancer at study entry, in order to include a reasonably representative study population. In addition, the BENCHMRK studies allowed patients to use investigational agents in their OBT.

A cumulative meta-analysis of safety was conducted including all available data from adult patients in the raltegravir clinical development program who received the 400 mg bid dose, thus including substantial additional raltegravir exposure beyond the phase III studies included in this report. This meta-analysis demonstrated a safety profile generally consistent with the STARTMRK and BENCHMRK studies, acknowledging the diversity of a population including both treatment-naïve and highly treatment-experienced patients.

In aggregate, data from the raltegravir clinical development program have demonstrated potent and durable efficacy in a broad patient population with HIV infection [2-6]. This manuscript provides an overview of the favorable safety profile established for raltegravir to date, and supports the use of this agent in combination regimens for the treatment of HIV infection. The safety of raltegravir will continue to be monitored in ongoing clinical trials, including studies in other patient populations such as pediatric patients, and by pharmacovigilance activities in the marketed environment, which include the continuous review of spontaneous reports of adverse experiences, and the conduct of prospective observational cohort studies.

## SUPPORTIVE/SUPPLEMENTARY MATERIAL

Cumulative meta-analysis: description of methods and results.Table: Patient demographics, meta-analysis populationTable: Summary of specific safety issues, meta-analysis populationTable: Liver function tests and hepatobiliary clinical events, meta-analysis population

## Figures and Tables

**Table 1:** Patient Demographics, Phase III Studies

	STARTMRK		BENCHMRK	

	Raltegravir N=281	Efavirenz N=282	Raltegravir N=462	Placebo N=237

**Gender**				

Female	19.2%	18.1%	12.3%	11.4%
Male	80.8%	81.9%	87.7%	88.6%

**Age (Years)**				

17 and Under	0.0%	0.0%	0.9%	0.4%
18 to 64	99.3%	98.6%	97.6%	98.7%
Over 64	0.7%	1.4%	1.5%	0.8%
Mean	37.6	36.9	45.7	45.1
Std. Dev.	8.97	9.98	8.56	8.12
Median	37.0	36.0	45.0	45.0
Range	19 - 67	19 - 71	16 - 74	17 - 70

**Race/Ethnicity**				

Asian	12.8%	11.3%	3.5%	2.5%
Black	11.7%	8.2%	14.1%	11.0%
Hispanic American	21.4%	23.8%	11.5%	8.0%
Multi-Racial	12.5%	12.8%	5.6%	5.5%
Native American	0.4%	0.4%	0.2%	0.0%
White	41.3%	43.6%	65.2%	73.0%

**Hepatitis Co-Infection**†				

Hepatitis B or C Positive	6.4%	5.7%	16.7%	15.6%

† Evidence of hepatitis B surface antigen or evidence of HCV RNA by polymerase chain reaction (PCR) quantitative test for hepatitic C Virus. In the STARTMRK study five patients previously classified as hepatitis B or C positive were subsequently identified based on lab tests as being hepatitis B or C negative. In the BENCHMRK -1 and -2 studies one patient from BENCHMRK-2 was included in the Hepatitis B or C positive subgroup due to HCV positive despite a negative HCV antibody as specified by the Food and Drug Administration.

**Table 2:** Summary of Clinical Adverse Experiences, Phase III Studies

Proportion of Patients	STARTMRK				BENCHMRK							

	Raltegravir N=281		Efavirenz N=282		Raltegravir N=462 PYR=708				Placebo N=237 PYR=244			

	%		%		%	Rate†			%			Rate†

**Clinical Adverse Experience (AE) Summary**												

With one or more AEs	94.3		97.2		92.4		60.3			88.6	86.1	
With drug-related‡ AEs	47.0		78.0		57.8		37.7			58.6	57.0	
With serious AEs	13.2		11.7		23.2		15.1			22.4	21.7	
With serious drug-related‡ AEs	2.1		1.8		2.8		1.8			3.8	3.7	
Who died	1.1		0.0		2.8		1.8			3.0	2.9	
Discontinued due to AEs	3.6		6.0		3.7		2.4			4.6	4.5	
Discontinued due to drug-related AEs	1.1		4.3		1.5		1.0			1.7	1.6	

**Clinical AEs by System Organ Class (Incidence ≥10%§) All Causality, All Intensities**												

**Gastrointestinal Disorders**												

Diarrhea	17.1		24.5		22.3		14.5			23.6	23.0	
Nausea	14.2		12.8		13.2		8.6			14.8	14.3	
Vomiting	6.8		8.9		8.0		5.2			10.5	10.2	

**General Disorders and Administrative Site Conditions**												

Fatigue	6.8		11.7		11.0		7.2			5.5	5.3	
Injection Site Reaction%	NA		NA		10.4		6.8			10.1	9.8	
Pyrexia	11.7		10.3		8.4		5.5			13.9	13.5	

**Infections and Infestations**												

Influenza	7.5		11.7		6.3		4.1			4.2	4.1	
Nasopharyngitis	17.4		13.5		11.3		7.3			6.8	6.6	
Upper Respiratory Tract Infections	14.6		15.2		13.0		8.5			8.9	8.6	

**Nervous System Disorder**												

Dizziness	8.2		36.9	5.6			3.7			2.5	2.5	
Headache	22.8		24.5	11.5			7.5			13.1	12.7	

**Psychiatric Disorders**												

Abnormal Dreams	7.5		13.1	0.9			0.6			1.3	1.2	
Insomnia	12.1		11.0	6.1			4.0			5.1	4.9	

**Respiratory, Thoracic and Mediastinal Disorders**												

Cough	12.1	9.2		6.3			4.1			5.9	5.7	

**Skin and Subcutaneous Tissue Disorders**												

Rash	6.0		12.1	7.6			4.9			3.8	3.7	

**Clinical AEs by System Organ Class (Incidence ≥2%§) Drug-Related¶ All Intensities**												

**Ear and Labyrinth Disorders**												
Vertigo	1.8		3.2	0.9			0.6			0.0	0.0	

**Gastrointestinal Disorders**												

Abdominal Distension	1.1	1.4		2.2				1.4	1.7		1.6	
Abdominal Pain	1.4	2.5		1.5				1.0	1.3		1.2	
Diarrhea	5.0	9.6		3.2				2.1	5.1		4.9	
Dyspepsia	1.4	2.1		0.6				0.4	0.0		0.0	
Flatulence	3.6	5.0		1.9				1.3	1.3		1.2	
Nausea	8.5	9.9		4.1				2.7	4.6		4.5	
Vomiting	1.4	4.6		1.5				1.0	2.1		2.0	

**General Disorders and Administrative Site Conditions**												

Asthenia	2.1	2.5		1.5				1.0	0.4		0.4	
Fatigue	3.9	8.5		3.2				2.1	0.8		0.8	
Pyrexia	1.4	1.8		0.9				0.6	2.5		2.5	

**Metabolism and Nutrition Disorders**												

Anorexia	1.4	3.2		0.0				0.0	0.8		0.8	

**Nervous System Disorders**												

Dizziness	6.0	34.0		1.3				0.8	0.4		0.4	
Headache	9.3	13.8		4.8				3.1	5.1		4.9	
Somnolence	1.1	7.4		0.6				0.4	0.8		0.8	

**Psychiatric Disorders**												

Abnormal Dreams	7.1	13.1		0.4				0.3	0.8		0.8	
Insomnia	6.4	7.4		1.5				1.0	0.8		0.8	
Nightmare	2.5	5.0		0.0				0.0	0.0		0.0	

**Skin and Subcutaneous Tissue Disorders**												

Rash	1.1	8.2		1.1				0.7	1.7		1.6	
Rash Maculo-Papular	0.0	3.2		0.6				0.4	0.4		0.4	

PYR = Person Years at Risk.

N = Number of patients in each treatment group.

NA – Not applicable. No injectable antiretroviral medications administered in STARTMRK study.

† Events per 100 person-years, with person-years at risk (PYR) calculated based on the overall endpoint.

‡ Determined by the investigator to be possibly, probably, or definitely drug-related.

¶ Determined by the investigator to be possibly, probably, or definitely drug-related to raltegravir/efavirenz (alone or in combination with OBT or tenofovir/emtricitabine.

§ Adverse Events presented in this table met the criteria for at least one parameter.

% All cases due to enfuvirtide injections.

Adverse Experience terms are from MedDRA Version 12.0.

Note: For BENCHMRK Studies Raltegravir and Placebo were administered with Optimized Background Therapy (OBT). For STARTMRK Raltegravir and Efavirenz were administered with tenofovir/emtricitabine.

**Table 3:** Grade 3 and 4 Laboratory Abnormalities, Phase III Studies†

Laboratory Test	Grade 3 Threshold	STARTMRK		BENCHMRK			
		Raltegravir N=281	Efavirenz N=282	Raltegravir N = 462 PYR = 708		Placebo N = 237 PYR = 244	
		%	%	%	Rate‡	%	Rate‡
Hemoglobin	<7.4 (gm/dL)	0.7	0.7	1.1	0.7	0.8	0.8
Absolute neutrophil count	<0.749 (10[3]/µL)	2.5	1.1	4.1	2.7	3.8	3.7
Platelet count	<49.999 (10[3]/µL)	0.0	0.4	1.3	0.8	0.9	0.8
Fasting LDL-C	≥190 (mg/dL)	1.1	6.5	5.8	2.8	6.5	4.5
Fasting cholesterol	>300 (mg/dL)	0.0	4.1	9.9	6.2	6.2	5.7
Fasting triglyceride	>751 (mg/dL)	0.4	1.5	9.9	6.2	6.7	6.1
Fasting glucose	>251 (mg/dL)	1.1	0.0	2.7	1.7	0.9	0.8
Total bilirubin	>2.6 x ULN (mg/dL)	0.7	0.0	3.7	2.4	2.5	2.5
Creatinine	≥1.9 x ULN (mg/dL)	0.0	0.0	1.7	1.1	1.3	1.2
Aspartate aminotransferase	>5.1 x ULN (IU/L)	2.8	2.5	4.8	3.1	4.2	4.1
Alanine aminotransferase	>5.1 x ULN (IU/L)	1.8	2.5	5.2	3.4	3.8	3.7
Alkaline phosphatase	>5.1 x ULN (IU/L)	0.0	0.7	1.1	0.7	1.7	1.6
Pancreatic amylase	>2.1 x ULN (IU/L)	NA	NA	3.9	2.5	3.4	3.3
Lipase	>3.1 x ULN (IU/L)	NA	NA	1.7	1.1	0.8	0.8
Creatine kinase	≥10.0 x ULN (IU/L)	NA	NA	6.7	4.4	3.4	3.3

PYR = Person Years at Risk.

N = Number of patients in each treatment group.

ULN = Upper Limit of Normal.

NA = Not Applicable. These tests not performed for STARTMRK study.

† For inclusion in this analysis, both a baseline and at least one on-treatment laboratory value had to be present. Only patients with a worsened grade from baseline were included. A patient was listed with a Grade X event if his/her highest grade during the treatment was X.

‡ Events per 100 person-years, with person-years at risk (PYR) calculated based on the overall endpoint.

Note: For BENCHMRK Studies Raltegravir and Placebo were administered with Optimized Background Therapy (OBT). For STARTMRK Raltegravir and Efavirenz were administered with tenofovir/emtricitabine.

**Table 4:** Summary of Specific Safety Issues, Phase III Studies

	STARTMRK					BENCHMRK							

	Raltegravir N=281	Efavirenz N=282				Raltegravir N=462; PYR=708			Placebo N=237; PYR=244				

	%	%				%	Rate†		%			Rate†	

**Rash in Overall Population**													

All	9.6	20.9				11.3		7.3	6.3			6.1	
Drug-related‡	1.8	13.8				2.6		1.7	3.8			3.7	
Serious	0.0	0.0				0.0		0.0	0.0			0.0	
Discontinued	0.0	1.1				0.0		0.0	0.0			0.0	

**Rash in Patients Who Received OBT with Darunavir**													

All	NA	NA				17.4		10.9		4.8		3.8	
Drug-related‡	NA	NA				3.9		2.4		2.9		2.3	
Serious	NA	NA				0.0		0.0		0.0		0.0	
Discontinued	NA	NA				0.0		0.0		0.0		0.0	

**Rash in Patients Who Received OBT without Darunavir**													

All	NA	NA			6.3			4.2		7.5			8.8
Drug-related‡	NA	NA			1.6			1.1		4.5			5.3
Serious	NA	NA			0.0			0.0		0.0			0.0
Discontinued	NA	NA			0.0			0.0		0.0			0.0

**Depression/Suicidality**													

All	7.5		8.9		3.7			2.4			5.5		5.3
Drug-related‡	2.5		3.9		0.6			0.4			0.8		0.8
Serious	0.7		0.7		0.4			0.3			0.8		0.8
Discontinued	0.0		0.4		0.0			0.0			0.0		0.0

**IRIS**													

All	6.8		4.6		0.4			0.3			0.4		0.4
Drug-related‡	3.2		1.1		0.0			0.0			0.4		0.4
Serious	1.8		0.7		0.4			0.3			0.0		0.0
Discontinued	0.4		0.0		0.0			0.0			0.0		0.0

**Lipodystrophy**													

All	0.0		0.7		5.6			3.7			3.0		2.9
Drug-related‡	0.0		0.4		4.8			3.1			3.0		2.9
Serious	0.0		0.0		0.0			0.0			0.0		0.0
Discontinued	0.0		0.0		0.0			0.0			0.0		0.0

**Increased CK with Clinical Manifestations (Myopathy, Myositis, or Rhabdomyolysis)**													

Grade 3 or 4 CK	NA			NA	6.7			4.4			3.4		3.3
Discontinued Due to Laboratory AE	0.0			0.0	0.0			0.0			0.0		0.0
Myopathy, Myositis or Rhabdomyolysis	0.4			0.0	0.2			0.1			0.4		0.4

PYR = Person Years at Risk.

N = Number of patients in each treatment group.

† Events per 100 person-years, with person-years at risk (PYR) calculated based on the overall endpoint.

‡ Determined by the investigator to be possibly, probably, or definitely drug related.

NA = Not Applicable.

Note: For BENCHMRK Studies Raltegravir and Placebo were administered with Optimized Background Therapy (OBT). For STARTMRK Raltegravir and Efavirenz were administered with tenofovir/ emtricitabine.

**Table 5:** Percent Change from Baseline in Whole Body Composition by DEXA in STARTMRK

	Raltegravir			Efavirenz		
Week	N	Baseline Mean (gm)	Mean % Change†(95% CI)	N	Baseline Mean (gm)	Mean % Change†(95% CI)
0	55	20408.54		56	17542.25	
48	40	20095.34	18.10 (6.22, 29.98)	46	17776.56	19.99 (12.42, 27.57)
96	37	21487.02	19.63 (3.26, 36.01)	38	17566.83	21.04 (12.16, 29.92)

N = Number of patients in the treatment group.

† Mean % change from baseline and are based on the measurements of the patients who were measured at baseline and the time point assessed.

The DEXA re-scan (for the baseline visit) values were taken as the baselines for 7 patients and clinically deemed acceptable, when the original baseline scan readings were not available.

Note: MK-0518 and Efavirenz were administered with tenofovir/emtricitabine.

**Table 6:** Liver Function Tests and Hepatobiliary Clinical Events, Phase III Studies

Adverse Experience (AE)	STARTMRK				BENCHMRK				

	Raltegravir N=281			Efavirenz N=282	Raltegravir N=462 PYR=708			Placebo N=237 PYR=244	

	%			%	%		Rate†	%	Rate†

**Overall Population: Liver Function Tests**									

Grade 3 or 4 ALT	1.8		2.5		5.2	3.4		3.8	3.7
Grade 3 or 4 AST	2.8		2.5		4.8	3.1		4.2	4.1
Grade 3 or 4 Total Bilirubin	0.7		0.0		3.7	2.4		2.5	2.5
Discontinued Due to Laboratory AE of ALT/AST/Bilirubin	0.0		0.7		0.2	0.1		0.0	0.0

**Overall Population: Hepatobiliary Clinical Events‡**									

All AEs	0.7		0.4		1.1		N/D	1.3	N/D
Drug-related§ AEs	0.4		0.4		0.9		N/D	1.3	N/D
Serious AEs	0.0		0.0		0.2		N/D	0.4	N/D
Discontinued Due to Clinical AEs	0.0		0.0		0.2		N/D	0.0	N/D

**Patients With Hepatitis B and/or C Co-Infection%: Liver Function Tests **									

Grade 3 or 4 ALT	5.6	12.5			13.0		8.0	8.1	9.1
Grade 3 or 4 AST	11.1	6.3			10.4		6.4	2.7	3.0
Grade 3 or 4 Total Bilirubin	0.0	0.0			3.9		2.4	5.4	6.0

**Patients With Hepatitis B and/or C Co-Infection%: Hepatobiliary Clinical Events**									

All AEs	0.0		0.0		1.3		N/D	0.0	N/D
Drug-related§ AEs	0.0		0.0		1.3		N/D	0.0	N/D
Serious AEs	0.0		0.0		0.0		N/D	0.0	N/D
Discontinued Due to Clinical AEs	0.0		0.0		0.0		N/D	0.0	N/D

**Patients Without Hepatitis B and/or C Co-Infection%: Liver Function Tests **									

Grade 3 or 4 ALT	1.5	1.9			3.6		2.4	3.0	2.9
Grade 3 or 4 AST	2.3	2.3			3.6		2.4	4.5	4.3
Grade 3 or 4 Total Bilirubin	0.8	0.0			3.6		2.4	2.0	1.9

**Patients Without Hepatitis B and/or C Co-infection%: Hepatobiliary Clinical Events**									

All AEs	0.8		0.4		1.0		N/D	1.5	N/D
Drug-related§ AE	0.4		0.4		0.8		N/D	1.5	N/D
Serious AE	0.0		0.0		0.3		N/D	0.5	N/D
Discontinued Due to Clinical AE	0.0		0.0		0.3		N/D	0.0	N/D

PYR = Person Years at Risk.

N = Number of patients in each treatment group.

† Events per 100 patient-years.

‡ Hepatobiliary Clinical Events include: acute hepatitic failure, hepatic cirrhosis, hepatic steatosis, hepatitis, hepatitis acute, hepatitis toxic, and jaundice.

§ Determined by the investigator to be possibly, probably, or definitely drug-related.

% Number of patients with Hepatitis B and/or C Infection: STARTMRK: Raltegravir = 18, Efavirenz = 16 and BENCHMRK: Raltegravir = 77, Placebo = 37. Number of patients without Hepatitis B and/or C Infection: STARTMRK: Raltegravir = 263, Efavirenz = 266 and BENCHMRK: Raltegravir = 385, Placebo = 200.

N/D = Not Done

**Table 7:** Summary of Clinical Adverse Experiences, Meta-Analysis Population

Proportion of Patients	Raltegravir 400 mg b.i.d. N=1298 PYR=1771			Comparators N=954 PYR=981			

	%	Rate†		%		Rate†	

**Clinical Adverse Experience (AE) Summary**							

With one of more AEs	84.8	62.2		78.6			76.5
With drug-related‡ AEs	42.2	30.9		48.6			47.3
With serious AEs	14.3	10.5		10.8			10.5
With serious drug-related‡ AEs	1.5	1.1		1.8			1.7
Who died	1.2	0.9		0.8			0.8
Discontinued due to AEs	2.6	1.9		3.6			3.5
Discontinued due to drug-related‡ AEs	0.9	0.7		2.2			2.1

**Clinical AEs by System Organ Class (Incidence ≥10%§) All Causality**,** All Intensities**							

**Gastrointestinal Disorders**							

Diarrhea	15.7	11.5		19.3		18.8	
Nausea	11.5	8.4		10.6		10.3	

**Infections and Infestations**							

Nasopharyngitis	10.8	7.9	7.4			7.2	
Upper Respiratory Tract Infection	12.1	8.9	9.4			9.2	

**Nervous System Disorders**							

Dizziness	6.0	4.4	13.3			12.9	
Headache	13.2	9.7	14.0			13.7	

**Clinical AEs by System Organ Class (Incidence ≥2%§) Drug-Related% All Intensities**							

**Gastrointestinal Disorders**							

Diarrhea	3.2	2.3	6.9		6.7		
Flatulence	2.3	1.7	2.6		2.5		
Nausea	5.2	3.8	5.7		5.5		
Vomiting	1.4	1.0	2.5		2.4		

**General Disorders and Administration Site Conditions**							

Fatigue	2.9	2.1	3.2		3.2		

**Nervous System Disorders**							

Dizziness	3.0	2.2	11.6		11.3		
Headache	5.6	4.1	6.9		6.7		
Somnolence	0.8	0.6	2.6		2.5		

**Psychiatric Disorders**							

Abnormal Dreams	2.5	1.8	4.8		4.7		
Insomnia	3.4	2.5	3.1		3.1		

**Skin and Subcutaneous Tissue Disorders**							

Rash	0.8	0.6	2.9		2.9		

PYR = Person Years at Risk.

N= Number of patients in each treatment group.

† Events per 100 patient-years.

‡ Determine by the investigator to be possibly, probably, or definitely drug-related.

§ Adverse Events presented in this table met the criteria for at lease one parameter.

% For Protocols 004, 005, 018, 019, and 021 determined by the investigator to be drug-related to raltegravir, efavirenz, or placebo (alone or in combination with ART). For Protocols 032 and 033 determined by the investigator to be drug-related or not related.

Adverse Experience terms are from MedDRA version 12.0 for Protocols 004, 005, 018, 019, and 021 and from MedDRA 11.1 for Protocols 032 and 033.

Note: Comparators include efavirenz in Protocols 004 and 021, placebo in Protocols 005, 018, and 019, lopinavir/ritonavir in Protocols 032 and 033.

**Table 8:** Grade 3 and 4 Laboratory Abnormalities, Meta-Analysis Population†

Laboratory Test	Grade 3 Threshold	Raltegravir 400 mg b.i.d. N=1298 PYR = 1771		Comparators N=954 PYR = 981	
		%	Rate‡	%	Rate‡
Hemoglobin	<7.4 (gm/dl)	0.5	0.4	0.4	0.4
Absolute neutrophil count	<0.749 (10[3]/microL)	2.2	1.6	1.5	1.4
Platelet count	<49.999 (10[3]/microL)	0.6	0.5	0.3	0.3
Fasting LDL-C	≥190 (mg/dL)	2.6	1.6	4.5	3.7
Fasting Cholesterol	>300 (mg/dL)	4.0	2.8	4.1	3.7
Fasting Triglyceride	>751 (mg/dL)	4.0	2.8	3.5	3.2
Fasting Glucose	>251 (mg/dL)	1.3	0.9	0.2	0.2
Total bilirubin	>2.6 (mg/dL)	1.9	1.4	1.2	1.1
Creatinine	≥1.9 x ULN (mg/dL)	0.7	0.5	0.4	0.4
Aspartate aminotransferase	>5.1 x ULN (IU/L)	3.1	2.3	2.1	2.0
Alanine aminotransferase	>5.1 x ULN (IU/L)	3.1	2.3	2.2	2.1
Alkaline phosphatase	>5.1 x ULN (IU/L)	0.5	0.3	0.6	0.6
Pancreatic amylase§	>2.1 x ULN (IU/L)	3.5	2.0	2.5	2.2
Lipase§	>3.1 x ULN (IU/L)	1.1	0.9	0.3	0.4
Creatine kinase§	≥10.0 x ULN (IU/L)	7.2	4.2	3.4	3.0

PYR = Person Years at Risk.

N = Number of patients in each treatment group.

ULN = Upper Limit of Normal.

† For inclusion in this analysis, both a baseline and at least one on-treatment laboratory value had to be present. Only patients with a worsened grade from baseline were included. A patient was listed with a Grade X event if his/her highest grade during treatment was X.

‡ Events per 100 person-years, with person-years at risk (PYR) calculated based on the overall endpoint.

§ Protocol 021 did not routinely collect pancreatic amylase, lipase, or creatine kinase. Protocols 032 and 033 did not routinely collect pancreatic amylase or lipase.

Note: Comparators include efavirenz in Protocols 004 and 021, placebo in Protocols 005, 018, and 019, lopinavir/ritonavir in Protocols 032 and 033.

## Footnotes

1 Miller M, Witmer M, Stillmock K, *et al*. Biochemical and antiviral activity of MK-0518, a potent HIV integrase inhibitor. Presented at: 16th International AIDS Conference; Toronto, Canada; August 2006 [Abstract THAA0302].

2 E. Gotuzzo, B.-Y. Nguyen, M. Markowitz, *et al*. Sustained Antiretroviral Efficacy of Raltegravir as part of Combination ART in Treatment-Naive HIV-1 infected patients: 144-week data [abstract]. Presented at the 5th Biennial IAS Conference on HIV Pathogenesis, Treatment and Prevention, July 2009.

3 Anderson MS, Sekar V, Tomaka F, *et al*. Pharmacokinetic (PK) Evaluation of Darunavir/Ritonavir (DRV/r) and Raltegravir (RAL) in Healthy Subjects. Presented at 48th Interscience Conference on Antimicrobial Agents and Chemotherapy, Oct 2008 [Abstract A-962].

4 Gatell J, Nguyen B-Y, Grinsztejn B, *et al*. 144-Week Efficacy and Safety of Raltegravir (RAL) in Treatment-Experienced Patients [abstract]. Presented at the 47th Annual Meeting of the Infectious Diseases Society of America, Philadelphia PA, October 2009.

## ABBREVIATIONS

ADC: = AIDS-defining conditions
AE: = Adverse event
ALT: = Alanine aminotransferase
AST: = Aspartate aminotransferase
CI: = Confidence interval
CK: = Creatine phosphokinase
DAIDS: = Division of AIDS
DB: = Double-blind
DEXA: = Dual energy x-ray absorptiometry
HAART: = Highly active antiretroviral therapy
HIV: = Human immunodeficiency virus
IRIS: = Immune reconstitution inflammatory syndrome
MedDRA: = Medical Dictionary for Drug Regulatory Affairs
NCI/ACS: = National Cancer Institute/American Cancer Society
OBT: = Optimized background therapy
PYR: = Person-years
RR: = Relative risk
SAE: = Serious adverse event
SJS: = Stevens-Johnson syndrome
