# Supplementary material for: Long-Term Safety from the Raltegravir Clinical Development Program
Source: Curr HIV Res. 2011 Jan;9(1):40–53. doi: 10.2174/157016211794582650 (PMC3267161; doi:10.2174/157016211794582650)
Supplement: Supplementary file 1 [file CHIVR-9-40_SD1.zip › Supplementary Material_3.doc]

**Table 2. Summary of Specific Safety Issues, Meta-Analysis Population**

|  | Raltegravir 400 mg b.i.d.  N=1298; PYR=1771 | | Comparators  N=954; PYR=981 | |
| --- | --- | --- | --- | --- |
|  | % | Rate^†^ | (%) | Rate^†^ |
| **Rash in Overall Population** | | | | |
| All | 7.9 | 5.8 | 9.7 | 9.5 |
| Drug-related^‡^ | 1.5 | 1.1 | 5.6 | 5.4 |
| Serious | 0.0 | 0.0 | 0.0 | 0.0 |
| Discontinued | 0.0 | 0.0 | 0.3 | 0.3 |
| **Depression/Suicidality** | | | | |
| All | 5.5 | 4.0 | 5.5 | 5.3 |
| Drug-related^‡^ | 1.0 | 0.7 | 1.4 | 1.3 |
| Serious | 0.7 | 0.5 | 0.4 | 0.4 |
| Discontinued | 0.1 | 0.1 | 0.1 | 0.1 |
| **IRIS** | | | | |
| All | 0.5 | 0.4 | 0.3 | 0.3 |
| Drug-related^‡^ | 0.2 | 0.1 | 0.2 | 0.2 |
| Serious | 0.5 | 0.4 | 0.2 | 0.2 |
| Discontinued | 0.1 | 0.1 | 0.0 | 0.0 |
| **Lipodystrophy** | | | | |
| All | 2.2 | 1.6 | 1.2 | 1.1 |
| Drug-related^‡^ | 1.9 | 1.4 | 1.0 | 1.0 |
| Serious | 0.0 | 0.0 | 0.1 | 0.1 |
| Discontinued | 0.0 | 0.0 | 0.1 | 0.1 |
| **Increased CK with Clinical Manifestations** (myopathy, myositis, or rhabdomyolysis) | | | | |
| Grade 3 or 4 CK | 7.2 | 4.2 | 3.4 | 3.0 |
| Discontinued Due to Laboratory AE | 0.0 | 0.0 | 0.0 | 0.0 |
| Myopathy, myositis, rhabdomyolysis | 0.2 | 0.2 | 0.1 | 0.1 |
| PYR = Person Years at Risk  N = Number of patients in each treatment group.  ^†^Events per 100 patient-years.  ^‡^For Protocols 004, 005, 018, 019, and 021 determined by the investigator to be possibly, probably, or definitely drug-related. For Protocols 032 and 033 determined by the investigator to be drug-related or not related.  Note: Comparators include efavirenz in Protocols 004 and 021, placebo in Protocols 005, 018, and 019, lopinavir and ritonavir in Protocols 032 and 033. | | | | |
