# Supplementary material for: Long-Term Safety from the Raltegravir Clinical Development Program
Source: Curr HIV Res. 2011 Jan;9(1):40–53. doi: 10.2174/157016211794582650 (PMC3267161; doi:10.2174/157016211794582650)
Supplement: Supplementary file 1 [file CHIVR-9-40_SD1.zip › Supplementary Material_4.doc]

**Table 3. Liver Function Tests and Hepatobiliary Clinical Events, Meta-Analysis**

**Population**

|  | Raltegravir 400 mg b.i.d.  N=1298; PYR=1771 | | Comparators  N=954; PYR=981 | |
| --- | --- | --- | --- | --- |
| Adverse Experience (AE) | % | Rate^†^ | % | Rate^†^ |
| **Overall Population: Liver Function Tests** | | | | |
| Grade 3 or 4 ALT | 3.1 | 2.3 | 2.2 | 2.1 |
| Grade 3 or 4 AST | 3.1 | 2.3 | 2.1 | 2.0 |
| Grade 3 or 4 Total Bilirubin | 1.9 | 1.4 | 1.2 | 1.1 |
| Discontinued Due to Laboratory AE of AST/ALT/Bilirubin | 0.4 | 0.3 | 0.2 | 0.2 |
| **Overall Population: Hepatobiliary Clinical Events^‡^** | | | | |
| All AEs | 0.9 | 0.7 | 0.4 | 0.4 |
| Drug-Related^§^ | 0.7 | 0.5 | 0.4 | 0.4 |
| Serious AEs | 0.1 | 0.1 | 0.1 | 0.1 |
| Discontinued Due to a Clinical AEs | 0.1 | 0.1 | 0.0 | 0.0 |
| PYR = Person Years at Risk.  N = Number of patients in each treatment group.  ^†^ Events per 100 patient-years.  **^‡^** Hepatobiliary Clinical Events include: acute hepatitic failure, hepatic cirrhosis, hepatic steatosis, hepatitis, hepatitis acute, hepatitis toxic, and jaundice.  Note: Comparators include efavirenz in Protocols 004 and 021, placebo in Protocols 005, 018, and 019, lopinavir and ritonavir in Protocols 032 and 033. | | | | |
