# Supplementary material for: Long-Term Safety from the Raltegravir Clinical Development Program
Source: Curr HIV Res. 2011 Jan;9(1):40–53. doi: 10.2174/157016211794582650 (PMC3267161; doi:10.2174/157016211794582650)
Supplement: Supplementary file 1 [file CHIVR-9-40_SD1.zip › Supplementary Material_1.doc]

**Methods**

In the cumulative meta-analysis, results are presented for clinical adverse experiences and for treatment emergent laboratory abnormalities for raltegravir 400 mg bid and for comparators (in aggregate). All studies and all populations were combined; only double blind treatment phase data were used. Although several of the included studies (P005 and BENCHMRK studies) allowed open label raltegravir treatment, for example as an option after virologic failure occurred, only the double blind phase data from those studies are included to permit appropriate comparison of randomized treatment groups. P004 was dose ranging (using 100 mg, 200 mg, 400 mg and 600 mg raltegravir bid) for the first 48 weeks, after which all patients receiving raltegravir were given the 400 mg bid dose. Since no dose related toxicities or differences in efficacy were observed in the first 48 weeks, all patients randomized to any dose of raltegravir in this study are included in the meta-analysis, thus incorporating an added 96 weeks of treatment at raltegravir dose of 400 mg bid. For protocol 005, only the patients randomized to the 400 mg raltegravir dose are included; the remaining studies used only the raltegravir 400 mg bid dose, thus all patients are included. The definition of a drug-related event from Protocols 004, 005, 018, 019, 021 is drug-related to raltegravir or comparator alone or in combination with other background therapy (OBT). The definition of a drug-related event from Protocol 032 and 033 is drug-related to any component of the study regimen (raltegravir, comparator, or OBT). MedDRA version 12.0 was used for all but Protocols 032 and 033, which used version 11.1. Criteria for serious adverse experiences and intensity were consistent among all studies; treatment emergent laboratory abnormalities were also handled consistently for this analysis and used the same logic as described above. Due to the noted imbalance in exposure in the BENCHMRK studies, exposure adjusted rates per 100 PYR as well as frequencies are provided, both for adverse experiences and treatment emergent laboratory abnormalities.

**Results**

In the supportive, cumulative meta-analysis of safety across the raltegravir adult clinical development program, baseline demographic characteristics were generally similar in patients receiving raltegavir and the comparator regimens (Table: 1). Overall trends for both number (%) and exposure-adjusted rates (1771 PYR for raltegravir vs 981 PYR for comparator) for all-causality, drug-related, serious, and serious and drug-related adverse experiences, as well as treatment emergent laboratory abnormalities, were broadly similar to those observed in the phase III studies as demonstrated by Tables 6 and 7 in the primary article and Tables 1-3 in this Appendix. No new safety concerns for raltegravir were identified.
