# Supplementary material for: Long-Term Safety from the Raltegravir Clinical Development Program
Source: Curr HIV Res. 2011 Jan;9(1):40–53. doi: 10.2174/157016211794582650 (PMC3267161; doi:10.2174/157016211794582650)
Supplement: Supplementary file 1 [file CHIVR-9-40_SD1.zip › Supplementary Material_2.doc]

**Table 1. Patient Demographics, Meta-Analysis Population**

|  | Raltegravir 400 mg b.i.d. | Comparators |
| --- | --- | --- |
| Demographics | N = 1298 | N = 954 |
| **Gender, n (%)** | | |
| Male | 1083 (83.4) | 777 (81.4) |
| Female | 215 (16.6) | 177 (18.6) |
| **Age (years)** | | |
| 17 and Under | 4 (0.3) | 1 (0.1) |
| 18 - 64 | 1277 (98.4) | 941 (98.6) |
| Over 64 | 17 (1.3) | 12 (1.3) |
| Mean (SD) | 42.1 (9.6) | 41.4 (9.6) |
| Median | 42.0 | 41.0 |
| Range | 16 to 74 | 17 to 74 |
| **Race/Ethnicity, n (%)** | | |
| Asian | 104 (8.0) | 79 (8.3) |
| Black | 165 (12.7) | 107 (11.2) |
| Hispanic American | 165 (12.7) | 102 (10.7) |
| Multiracial/Other | 132 (10.2) | 103 (10.8) |
| White | 732 (56.4) | 563 (59.0) |
